# *tert*-Butyl 1-hy­droxy­piperidine-2-carboxyl­ate

**DOI:** 10.1107/S1600536811026894

**Published:** 2011-07-16

**Authors:** Oliver Brücher, Uwe Bergsträsser, Harald Kelm, Jens Hartung

**Affiliations:** a Fachbereich Chemie, Organische Chemie, Technische Universität Kaiserslautern, Erwin-Schrödinger-Strasse, D-67663 Kaiserslautern, Germany; b Fachbereich Chemie, Anorganische Chemie, Technische Universität Kaiserslautern, Erwin-Schrödinger-Strasse, D-67663 Kaiserslautern, Germany

## Abstract

The title compound, C_10_H_19_NO_3_, is a disubstituted piperidine bearing substituents in two equatorial positions. One of the substituents is a hy­droxy group bound to nitro­gen and the second a *tert*-butyl ester group bound to the carbon next to the endocyclic nitro­gen. Enanti­omers of the title compound form hydrogen-bridged dimers across a center of inversion.

## Related literature

For bond lengths, see: Allen *et al.* (1987 ▶). For structural features associated with hydroxyl­amine, see: Chung-Phillips & Jebber (1995 ▶). For details of vanadium(V)- and molybdenum(VI)-catalysed oxidations, see: Hartung & Greb (2002 ▶); Reinhardt (2006 ▶). For a related structure, see: Kliegel *et al.* (2002 ▶). For the synthesis of 1-hy­droxy piperidine-2-carboxyl­ic acid, see: Murahashi & Shiota (1987 ▶). 
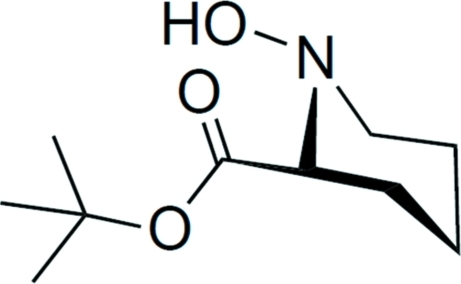


## Experimental

### 

#### Crystal data

C_10_H_19_NO_3_
*M* *_r_* = 201.26
Monoclinic, 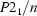

*a* = 10.1685 (3) Å
*b* = 12.1271 (2) Å
*c* = 10.2083 (3) Å
β = 110.377 (3)°
*V* = 1180.06 (5) Å^3^
*Z* = 4
Cu *K*α radiation
μ = 0.68 mm^−1^
*T* = 150 K
0.24 × 0.21 × 0.19 mm

#### Data collection

Oxford Diffraction Gemini S Ultra diffractometer
Absorption correction: multi-scan (*CrysAlis RED*; Oxford Diffraction, 2008 ▶) *T*_min_ = 0.854, *T*_max_ = 0.882
5815 measured reflections
1851 independent reflections
1440 reflections with *I* > 2σ(*I*)
*R*_int_ = 0.026

#### Refinement

*R*[*F* ^2^ > 2σ(*F* ^2^)] = 0.049
*wR*(*F* ^2^) = 0.146
*S* = 1.09
1851 reflections
131 parameters
H-atom parameters constrained
Δρ_max_ = 0.39 e Å^−3^
Δρ_min_ = −0.23 e Å^−3^

### 

Data collection: *CrysAlis CCD* (Oxford Diffraction, 2008 ▶); cell refinement: *CrysAlis RED* (Oxford Diffraction, 2008 ▶); data reduction: *CrysAlis RED*; program(s) used to solve structure: *SHELXS97* (Sheldrick, 2008 ▶); program(s) used to refine structure: *SHELXL97* (Sheldrick, 2008 ▶); molecular graphics: *ORTEP-3* (Farrugia, 1997) ▶; software used to prepare material for publication: *SHELXL97*.

## Supplementary Material

Crystal structure: contains datablock(s) I, global. DOI: 10.1107/S1600536811026894/nc2235sup1.cif

Structure factors: contains datablock(s) I. DOI: 10.1107/S1600536811026894/nc2235Isup2.hkl

Supplementary material file. DOI: 10.1107/S1600536811026894/nc2235Isup3.cml

Additional supplementary materials:  crystallographic information; 3D view; checkCIF report

## Figures and Tables

**Table 1:** Hydrogen-bond geometry (Å, °)

*D*—H⋯*A*	*D*—H	H⋯*A*	*D*⋯*A*	*D*—H⋯*A*
O3—H3⋯N1^i^	0.84	2.12	2.8136 (19)	139

Symmetry code: (i) 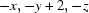.

**Fig. 1.**
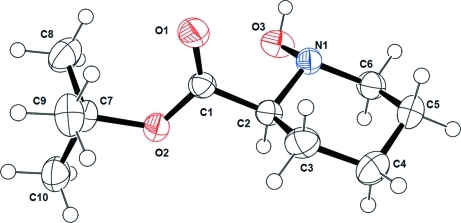
Molecular structure of title compound in the solid state. Displacement ellipsoids are drawn at the 50% probability level.

**Fig. 2.**
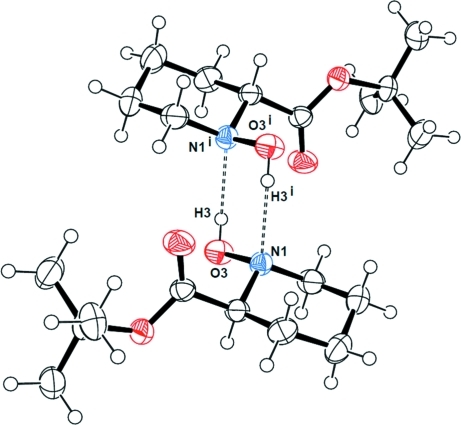
H-bridged dimers of title compound in the solid state [hydrogen bonds shown as dashed lines symmetry code: (i) -x, -y + 2, -z].

## supplementary crystallographic information

### Comment

1-Hydroxypiperidine-2-carboxylate (Murahashi & Shiota, 1987) attracted our attention, because the compound was expected to bind as dianion to early transition metal ions, such as vanadium(V) or molybdenum(VI). Complexes formed from vanadium(V) or molybdenum(VI) ions are formaly d^0^-metal centers. Complexes having such an electron configuration are able to activate peroxides at low temperatures, which is of importance for modern sustainable oxidation catalysis, for example in natural product synthesis (Hartung & Greb, 2002) or bleaching (Reinhardt, 2006). Since impurities from the synthesis of 1-hydroxypiperidine-2-carboxylate were difficult to separate from standard liquid/liquid and liquid/solid partitioning processes, we chose to convert this acid into a derived *O*-*tert*-butyl ester for subsequent sublimination. Colorless crystals of the title compound that deposited from the sublimation process were investigated *via* X-ray diffraction, in order to obtain a deeper structural insight into the product class of *N*-hydroxy α-aminocarboxylic acid esters.

The central structural element of the title compound, is a disubstituted piperidine ring. The N-heterocycle bears a hydroxy substituent at nitrogen and a *tert*-butyl O-ester substituent at the carbon next to the endocyclic nitrogen. Both substituents are bond to equatorial sites in piperidine (Figure 1). A distorted *gauche* arrangement of subunits C6–N1–O3–H3 = -90.30 ° and C2–N1–O3–H3 151.18 °, in combination with a N1–O3 distance of 1.4477 (18) Å, reflect typical structural characteristics of with compounds having a nitrogen oxygen single bond, such as hydroxylamine (Chung-Phillips & Jebber, 1995) or *N*-hydroxypiperidinium chloride (Kliegel *et al.*, 2002). The geometrical parameters for the *tert*-butyl O-ester group in terms of bond distances and angles agree with reference data reported previously (Allen *et al.*, 1987).

In the crystal, association of the title compound, occurs predominantly *via* H-bonding. Enantiomers of the title compound thus form H-bridged dimers (Figure 2 and Table 1) across a center of inversion [N1^i^···H3–O3 = 2.12 Å, N1^i^···O3 = 2.8136 (19)].

### Experimental

To a suspension of crude *N*-hydroxypiperidine-2-carboxylic acid (1.15 g, 8 mmol) (Murahashi & Shiota, 1987) in *tert*-butyl acetate (20 ml) was added at 298 K HClO_4_ [0.2 ml, 70% (*w*/*w*)]. The mixture was stirred for 10 min at 298 K and treated with a second batch of HClO_4_ [2 ml, 70% (*w*/*w*)]. Stirring was continued for 20 h at 298 K. pH of the reaction mixture was adjusted to 8–9 by addition of satd. aq. NaHCO_3_ [150 ml, 10% (*w*/*w*)] and NaOH pellets (0.8 g, 20 mmol). The resulting mixture was extracted with EtOAc (4 *x* 40 ml). Combined organic washings were dried (MgSO_4_) and concentrated under reduced pressure to furnish a brown oily residue that was purified by chromatography [SiO_2_, pentane/EtOAc = 2:1 (*v*/*v*)]. Yield: 412 mg (25%); mp 356 K; ^1^H NMR (600 MHz, CDCl_3_, δ_H_ p.p.m.): 1.20–1.30 (m, 1H), 1.47 (s, 9H), 1.52–1.78 (m, 4H), 1.97 (d, J = 11.1 Hz, 1H), 2.52 (t, J = 9.1 Hz, 1H), 3.02 (d, J = 10.6 Hz, 1H), 3.37 (d, J = 10.2 Hz, 1H), 6.56 (br s, 1H, OH). ^13^C NMR (151 MHz, CDCl_3_, δ_C_ p.p.m.): 23.1, 25.1, 28.0, 29.3, 57.4, 71.2, 81.2, 171.9. Anal. calcd. for C_15_H_23_NO_2_: C, 59.68; H, 9.52; N, 6.96%; Found C, 59.96; H, 9.49; N 6.94%. Crystalls suitable for X-ray diffraction were obtained by slow sublimation of (I) at 340 K and 0.1 mbar.

### Refinement

All H atoms were positioned geometrically and treated as riding atoms, with C—H distances in the range 0.98–1.00Å and with *U*_iso_(H) set at 1.2*U*_eq_ (CH_2_, CH) or 1.5*U*_eq_ (CH_3_ and OH) of the parent atom. A free rotating group refinement was used for CH_3_ and OH H atoms.

### Crystal data

**Table:**

C_10_H_19_NO_3_	*F*(000) = 440
*M**_r_* = 201.26	*D*_x_ = 1.133 Mg m^−^^3^
Monoclinic, *P*2_1_/*n*	Melting point: 356 K
Hall symbol: -P 2yn	Cu *K*α radiation, λ = 1.54184 Å
*a* = 10.1685 (3) Å	Cell parameters from 2751 reflections
*b* = 12.1271 (2) Å	θ = 3.6–62.6°
*c* = 10.2083 (3) Å	µ = 0.68 mm^−^^1^
β = 110.377 (3)°	*T* = 150 K
*V* = 1180.06 (5) Å^3^	Indifferent fragment, colourless
*Z* = 4	0.24 × 0.21 × 0.19 mm

### Data collection

**Table:**

Oxford Diffraction Gemini S Ultra diffractometer	1851 independent reflections
Radiation source: fine-focus sealed tube	1440 reflections with *I* > 2σ(*I*)
graphite	*R*_int_ = 0.026
Detector resolution: 16.1399 pixels mm^-1^	θ_max_ = 62.7°, θ_min_ = 6.4°
ω–scans	*h* = −11→11
Absorption correction: multi-scan (*CrysAlis RED*; Oxford Diffraction, 2008)	*k* = −13→12
*T*_min_ = 0.854, *T*_max_ = 0.882	*l* = −11→11
5815 measured reflections	

### Refinement

**Table:**

Refinement on *F*^2^	Primary atom site location: structure-invariant direct methods
Least-squares matrix: full	Secondary atom site location: difference Fourier map
*R*[*F*^2^ > 2σ(*F*^2^)] = 0.049	Hydrogen site location: inferred from neighbouring sites
*wR*(*F*^2^) = 0.146	H-atom parameters constrained
*S* = 1.09	*w* = 1/[σ^2^(*F*_o_^2^) + (0.1003*P*)^2^] where *P* = (*F*_o_^2^ + 2*F*_c_^2^)/3
1851 reflections	(Δ/σ)_max_ = 0.001
131 parameters	Δρ_max_ = 0.39 e Å^−^^3^
0 restraints	Δρ_min_ = −0.23 e Å^−^^3^

### Special details

**Table:**

Experimental. CrysAlis RED, Oxford Diffraction Ltd., (Version 1.171.31.8) Empirical absorption correction using spherical harmonics,implemented in SCALE3 ABSPACK scaling algorithm.
Geometry. All e.s.d.'s (except the e.s.d. in the dihedral angle between two l.s. planes) are estimated using the full covariance matrix. The cell e.s.d.'s are taken into account individually in the estimation of e.s.d.'s in distances, angles and torsion angles; correlations between e.s.d.'s in cell parameters are only used when they are defined by crystal symmetry. An approximate (isotropic) treatment of cell e.s.d.'s is used for estimating e.s.d.'s involving l.s. planes.
Refinement. Refinement of *F*^2^ against ALL reflections. The weighted *R*-factor *wR* and goodness of fit *S* are based on *F*^2^, conventional *R*-factors *R* are based on *F*, with *F* set to zero for negative *F*^2^. The threshold expression of *F*^2^ > σ(*F*^2^) is used only for calculating *R*-factors(gt) *etc*. and is not relevant to the choice of reflections for refinement. *R*-factors based on *F*^2^ are statistically about twice as large as those based on *F*, and *R*- factors based on ALL data will be even larger.

### Fractional atomic coordinates and isotropic or equivalent isotropic displacement parameters (Å^2^)

**Table:**

	*x*	*y*	*z*	*U*_iso_*/*U*_eq_	
C1	0.22238 (18)	0.85238 (14)	−0.04530 (19)	0.0323 (4)	
O1	0.11112 (15)	0.80571 (12)	−0.08516 (16)	0.0512 (5)	
O2	0.32151 (13)	0.84628 (10)	−0.10296 (13)	0.0368 (4)	
C2	0.27379 (18)	0.92188 (14)	0.08639 (18)	0.0330 (4)	
H2	0.3532	0.9699	0.0850	0.040*	
N1	0.15807 (15)	0.98997 (12)	0.09477 (14)	0.0313 (4)	
O3	0.12723 (13)	1.06522 (10)	−0.02218 (14)	0.0401 (4)	
H3	0.0425	1.0839	−0.0481	0.060*	
C3	0.3223 (2)	0.84527 (16)	0.2126 (2)	0.0439 (5)	
H3A	0.2449	0.7946	0.2093	0.053*	
H3B	0.4019	0.8002	0.2085	0.053*	
C4	0.3675 (2)	0.9091 (2)	0.3492 (2)	0.0541 (6)	
H4A	0.4526	0.9528	0.3590	0.065*	
H4B	0.3901	0.8572	0.4289	0.065*	
C5	0.2493 (2)	0.9855 (2)	0.3503 (2)	0.0529 (6)	
H5A	0.1686	0.9411	0.3532	0.063*	
H5B	0.2814	1.0323	0.4351	0.063*	
C6	0.2036 (2)	1.05791 (16)	0.2217 (2)	0.0427 (5)	
H6A	0.1254	1.1060	0.2230	0.051*	
H6B	0.2826	1.1056	0.2217	0.051*	
C7	0.3043 (2)	0.77301 (16)	−0.22421 (19)	0.0386 (5)	
C8	0.1799 (2)	0.8114 (2)	−0.3490 (2)	0.0554 (6)	
H8A	0.1910	0.8897	−0.3664	0.083*	
H8B	0.0931	0.8008	−0.3292	0.083*	
H8C	0.1755	0.7683	−0.4317	0.083*	
C9	0.2929 (3)	0.65452 (17)	−0.1852 (2)	0.0519 (6)	
H9A	0.2054	0.6440	−0.1668	0.078*	
H9B	0.3728	0.6360	−0.1012	0.078*	
H9C	0.2932	0.6065	−0.2623	0.078*	
C10	0.4397 (2)	0.79292 (19)	−0.2509 (2)	0.0509 (6)	
H10A	0.5193	0.7704	−0.1688	0.076*	
H10B	0.4481	0.8715	−0.2691	0.076*	
H10C	0.4395	0.7498	−0.3322	0.076*	

### Atomic displacement parameters (Å^2^)

**Table:**

	*U*^11^	*U*^22^	*U*^33^	*U*^12^	*U*^13^	*U*^23^
C1	0.0292 (9)	0.0312 (9)	0.0348 (10)	0.0023 (7)	0.0091 (8)	−0.0018 (8)
O1	0.0362 (8)	0.0574 (9)	0.0610 (10)	−0.0095 (7)	0.0185 (7)	−0.0249 (7)
O2	0.0354 (7)	0.0415 (7)	0.0341 (7)	−0.0039 (5)	0.0131 (6)	−0.0072 (5)
C2	0.0282 (9)	0.0354 (9)	0.0346 (10)	0.0003 (7)	0.0099 (7)	−0.0031 (8)
N1	0.0323 (8)	0.0312 (8)	0.0297 (8)	0.0030 (6)	0.0100 (6)	0.0012 (6)
O3	0.0359 (7)	0.0372 (7)	0.0476 (8)	0.0030 (5)	0.0151 (6)	0.0163 (6)
C3	0.0411 (11)	0.0414 (10)	0.0452 (12)	0.0090 (8)	0.0097 (9)	0.0070 (9)
C4	0.0517 (12)	0.0692 (13)	0.0336 (11)	0.0100 (11)	0.0052 (9)	0.0044 (10)
C5	0.0500 (12)	0.0755 (14)	0.0312 (11)	0.0035 (11)	0.0117 (9)	−0.0094 (10)
C6	0.0361 (10)	0.0424 (11)	0.0490 (12)	−0.0018 (8)	0.0144 (9)	−0.0150 (9)
C7	0.0433 (11)	0.0444 (10)	0.0280 (9)	−0.0017 (8)	0.0120 (8)	−0.0086 (8)
C8	0.0564 (14)	0.0666 (14)	0.0355 (11)	−0.0055 (11)	0.0063 (10)	−0.0027 (10)
C9	0.0704 (15)	0.0476 (12)	0.0461 (12)	−0.0023 (10)	0.0307 (11)	−0.0073 (10)
C10	0.0546 (13)	0.0609 (13)	0.0411 (11)	−0.0055 (10)	0.0217 (10)	−0.0095 (10)

### Geometric parameters (Å, °)

**Table:**

C1—O1	1.202 (2)	C5—H5A	0.9900
C1—O2	1.335 (2)	C5—H5B	0.9900
C1—C2	1.517 (2)	C6—H6A	0.9900
O2—C7	1.484 (2)	C6—H6B	0.9900
C2—N1	1.464 (2)	C7—C9	1.506 (3)
C2—C3	1.524 (3)	C7—C10	1.514 (3)
C2—H2	1.0000	C7—C8	1.522 (3)
N1—O3	1.4477 (18)	C8—H8A	0.9800
N1—C6	1.468 (2)	C8—H8B	0.9800
O3—H3	0.8400	C8—H8C	0.9800
C3—C4	1.520 (3)	C9—H9A	0.9800
C3—H3A	0.9900	C9—H9B	0.9800
C3—H3B	0.9900	C9—H9C	0.9800
C4—C5	1.521 (3)	C10—H10A	0.9800
C4—H4A	0.9900	C10—H10B	0.9800
C4—H4B	0.9900	C10—H10C	0.9800
C5—C6	1.512 (3)		
			
O1—C1—O2	126.22 (16)	H5A—C5—H5B	108.1
O1—C1—C2	123.69 (16)	N1—C6—C5	110.36 (16)
O2—C1—C2	109.97 (14)	N1—C6—H6A	109.6
C1—O2—C7	120.89 (13)	C5—C6—H6A	109.6
N1—C2—C1	109.21 (13)	N1—C6—H6B	109.6
N1—C2—C3	108.94 (15)	C5—C6—H6B	109.6
C1—C2—C3	108.69 (15)	H6A—C6—H6B	108.1
N1—C2—H2	110.0	O2—C7—C9	110.40 (15)
C1—C2—H2	110.0	O2—C7—C10	101.79 (14)
C3—C2—H2	110.0	C9—C7—C10	110.98 (17)
O3—N1—C2	104.77 (12)	O2—C7—C8	109.72 (16)
O3—N1—C6	106.58 (13)	C9—C7—C8	113.25 (18)
C2—N1—C6	110.82 (13)	C10—C7—C8	110.10 (17)
N1—O3—H3	109.5	C7—C8—H8A	109.5
C4—C3—C2	111.74 (16)	C7—C8—H8B	109.5
C4—C3—H3A	109.3	H8A—C8—H8B	109.5
C2—C3—H3A	109.3	C7—C8—H8C	109.5
C4—C3—H3B	109.3	H8A—C8—H8C	109.5
C2—C3—H3B	109.3	H8B—C8—H8C	109.5
H3A—C3—H3B	107.9	C7—C9—H9A	109.5
C3—C4—C5	109.27 (17)	C7—C9—H9B	109.5
C3—C4—H4A	109.8	H9A—C9—H9B	109.5
C5—C4—H4A	109.8	C7—C9—H9C	109.5
C3—C4—H4B	109.8	H9A—C9—H9C	109.5
C5—C4—H4B	109.8	H9B—C9—H9C	109.5
H4A—C4—H4B	108.3	C7—C10—H10A	109.5
C6—C5—C4	110.60 (17)	C7—C10—H10B	109.5
C6—C5—H5A	109.5	H10A—C10—H10B	109.5
C4—C5—H5A	109.5	C7—C10—H10C	109.5
C6—C5—H5B	109.5	H10A—C10—H10C	109.5
C4—C5—H5B	109.5	H10B—C10—H10C	109.5
			
O1—C1—O2—C7	−3.0 (3)	C1—C2—C3—C4	−176.65 (16)
C2—C1—O2—C7	173.14 (14)	C2—C3—C4—C5	54.3 (2)
O1—C1—C2—N1	−42.6 (2)	C3—C4—C5—C6	−53.9 (3)
O2—C1—C2—N1	141.07 (14)	O3—N1—C6—C5	−175.52 (14)
O1—C1—C2—C3	76.1 (2)	C2—N1—C6—C5	−62.1 (2)
O2—C1—C2—C3	−100.19 (17)	C4—C5—C6—N1	58.1 (2)
C1—C2—N1—O3	−65.86 (16)	C1—O2—C7—C9	−61.7 (2)
C3—C2—N1—O3	175.55 (13)	C1—O2—C7—C10	−179.57 (16)
C1—C2—N1—C6	179.56 (14)	C1—O2—C7—C8	63.8 (2)
C3—C2—N1—C6	60.98 (19)	C2—N1—O3—H3	152.19
N1—C2—C3—C4	−57.7 (2)	C6—N1—O3—H3	−90.30

### Hydrogen-bond geometry (Å, °)

**Table:**

*D*—H···*A*	*D*—H	H···*A*	*D*···*A*	*D*—H···*A*
O3—H3···N1^i^	0.84	2.12	2.8136 (19)	139

Symmetry codes: (i) −*x*, −*y*+2, −*z*.
